# The prognostic utility of GRACE risk score in predictive adverse cardiovascular outcomes in patients with NSTEMI and multivessel disease

**DOI:** 10.1186/s12872-022-03025-6

**Published:** 2022-12-26

**Authors:** Xiaokang Chen, Hao Wu, Liangpeng Li, Xiaofang Zhao, Chao Zhang, Wei Eric Wang

**Affiliations:** 1 Department of Cardiology, Daping Hospital, Third Military Medical University (Army Medical University), 10 Changjiang Branch Road, Chongqing, 400042 China; 2 Department of Cardiology, Santai County People’s Hospital (Affiliated Hospital of North Sichuan Medical College in Santai County), Mianyang, 621100 China

**Keywords:** GRACE score, NSTEMI, MVD, Long-term prognosis

## Abstract

**Background:**

GRACE risk score models are capable of predicting all-cause mortality of non-ST elevation myocardial infarction (NSTEMI) patients. However, its utility for evaluating major adverse cardiovascular events (MACE) in NSTEMI patients with multivessel disease (MVD) remains unclear.

**Methods and results:**

This study was designed as a retrospective cohort study that recruited patients with NSTEMI and multivessel disease between September 2013 and December 2018 in Daping Hospital, Chongqing, China. The primary outcome was a composite outcome that included all-cause mortality, recurrent angina, non-fatal myocardial infarction, coronary re-vascularization, and non-fatal strokes. Of the 827 patients with NSTEMI, 32 did not complete follow-up and 430 were excluded because of single-vessel disease. The remaining 365 NSTEMI patients with MVD had a median follow-up of 3.0 (IQR 2.6–3.3) years, 78 patients experienced outcomes. The GRACE risk score predicted the MACE (hazard ratio 1.014, 95% CI 1.006–1.021, *P* < 0.001). The GRACE risk score performed well in predicting all-cause mortality (*c*-statistic 0.72, 95% CI 0.59–0.85, *P* = 0.001) in MVD but was less powerful in predicting MACE (*c*-statistic 0.69, 95% CI 0.62–0.75, *P* < 0.001). When combining the GRACE risk score with the SYNTAX score, and blood urea nitrogen for predicting all-cause mortality and MACE events, the *c*-statistic value increased to 0.82 and 0.81 (*P* < 0.001).

**Conclusion:**

In NSTEMI patients with MVD, the GRACE score showed an acceptable predictive value for all-cause mortality, but it was less powerful in predicting MACE. Blood urea nitrogen may be valuable in assessing long-term cardiovascular events in patients with MVD.

**Supplementary Information:**

The online version contains supplementary material available at 10.1186/s12872-022-03025-6.

## Introduction

Multivessel disease (MVD) was encountered in approximately 50% of patients with non-ST-segment elevation myocardial infarction (NSTEMI) [1–3]. Compared with single vessel disease, NSTEMI patients with multivessel disease have reported with a higher incidences of cardiovascular events [4]. In combination with advancing age and comorbidities, their risks for major adverse cardiovascular events (MACE) were substantially increased [4, 5]. Compared with STEMI and stable coronary artery disease, there was currently less evidence describing the optimal treatment strategy for NSTEMI [4]. Therefore, it was prudent important to carry out risk assessment for the long-term prognosis of NSTEMI patients with MVD.

The GRACE risk score provided an excellent discriminative performance among risk assessment models with all-cause mortality as the clinical endpoint [6–8]. It was also recommended by 2020 European Society of Cardiology guidelines of non-ST-segment elevation acute coronary syndrome management to assess short- or long-term mortality risk in patients with NSTEMI [9]. The GRACE risk score was originally developed to estimate the risk of death in hospitals, and the clinical endpoint only takes into account all-cause mortality [9], excluding other cardiovascular events such as recurrent angina, non-fatal myocardial infarction, coronary revascularization. But these diseases also provided great burdens to the public health and economics. And until now, the ideal tool predicting the incidence of the MACE of NSTEMI patients was still lacking, and whether the GRACE score was suitable for assessing MACE of NSETMI patients remained unclear, especially in patients with MVD.

To solve the current dilemma, we used the clinical data of NSTEMI patients with MVD, which were regularly followed up for 3 years, to analyze the factors influencing the long-term prognosis of patients and the predictive discriminatory capacity of GRACE score.

## Methods

### Study population

This study was designed as a retrospective registry of patients admitted with the diagnosis of acute NSTEMI in Daping Hospital (tertiary medical center with emergency departments in Chongqing, China) of the Army Military Medical University from September 2013 to December 2018 (According to the criteria recommended by 2011/2015 European Society of Cardiology Guidelines [10, 11]). Based on the results of coronary angiography, NSTEMI patients with MVD were enrolled. Multivessel coronary artery disease was defined as a lesion ≥ 70% in at least 2 native coronary artery distributions. The patients were followed up for 3 years from the date of diagnosed as MVD. Patients’ status was checked from medical records in hospitals or by telephone for any patients who had moved during the follow-up. The outcomes were the major adverse cardiovascular events (MACE) of all-cause mortality included in-hospital mortality, recurrent angina, nonfatal MI, coronary revascularization, and nonfatal stroke. The study was reviewed and approved by the Ethics Committee of Daping Hospital. All patients provided written informed consent, and this study was conducted in accordance with the Declaration of Helsinki.

### General clinical information

The following data were collected from the electronic medical records: age, gender, weight, height, previous medical history, medications on admission, heart rate and blood pressure on admission, cardiac arrest at admission, Killip classification, 12 Lead electrocardiogram, cardiac ultrasound report, time of onset of symptoms, time of first medical contact, time of coronary angiography, length of hospital stay, and routinely available laboratory data including potassium, serum creatinine (Scr), blood urea nitrogen (BUN), fasting glucose, creatine kinase-MB, Troponin I, total cholesterol, low-density lipoprotein cholesterol, high-density lipoprotein cholesterol, triglycerides, and full blood count. If a patient had been checked multiple times, only the first measurement result was taken.

The coronary angiography report was confirmed by the experienced interventional cardiologist in a blinded manner. The analyzed coronary angiography data included severity of coronary artery stenosis (left main artery, left anterior descending artery, circumflex artery, right coronary artery), culprit vessel if applicable, stent type, and Thrombolysis in Myocardial Infarction (TIMI) classification. We used the widely accepted methods to calculate the following three scoring systems: TIMI, Global Registry of Acute Coronary Events (GRACE) [9], and Synergy between PCI with Taxus and cardiac surgery (SYNTAX) score [12]. All the scoring systems were assessed at the time of the patients admitted to the emergency department.

### Statistical analysis

The data were presented as median and interquartile ranges or percentages, unless otherwise indicated. Baseline characteristics were compared according to the quartiles of the GRACE risk score [Quartile 1 (≤ 122); Quartile 2 (123–143); Quartile 3 (144–165); Quartile 4 (≥ 166)]. Comparisons among multiple groups were performed using the Kruskal–Wallis H test or χ^2^ test according to their variable types. We used Cox proportional hazards regression models to investigate the association between the MACE and GRACE risk score values, and the results were expressed as hazard ratios (HR) and 95% confidence intervals (CI). A multivariate adjustment method was used to control for confounding factors: based on the significance of univariate regression analysis (*P* < 0.05) or their biological plausibility (i.e., heart rate, hypertension, chronic kidney disease, and Previous MI), these covariates were selected as potential confounding factors and forced into the multivariate Cox model analyses. Every 10-point increment in the GRACE score was calculated by GRACE score divided by 10. We used Kaplan-Meier plots to display the cumulative risk of MACE, and a log-rank test was used to compare groups. To assess the discriminatory capacity of cardiovascular events, receiver operating characteristic curve analysis was performed, and results were expressed as *c*-statistic. The data were analyzed by SPSS 25.0 software (New York, USA). The value of two-sided *P* < 0.05 was considered statistically significant.

## Results

### Characteristics of patients

**Fig. 1:**
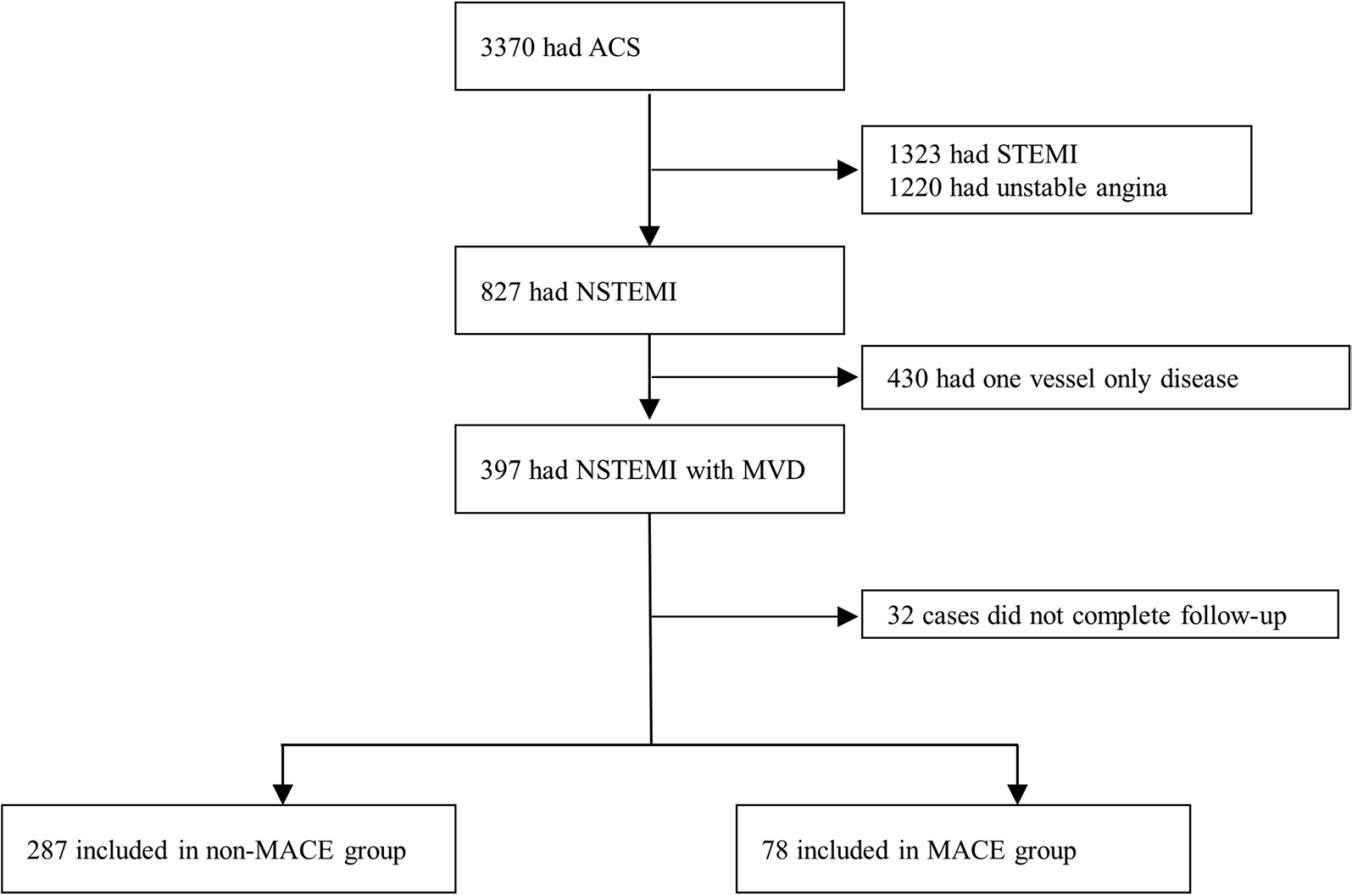
Study profile

Of the 3370 patients who had acute coronary syndrome in the study period September 2013 to December 2018, 397 (11.8%) patients had a diagnosis of NSTEMI with MVD. 32(0.08%) patients were excluded because of missing follow-up data. Finally,365 NSTEMI patients (278 men and 87 women; age, 65.5 ± 11.0 years) were included in the analyze. During median 3.0(IQR 2.6–3.3) years follow-up 78(21.4%) patients experienced outcomes. These included 18(4.9%) all-cause mortality, 18(4.9%) recurrent angina, 15(4.1%) nonfatal MI, 34(9.3%) coronary revascularization, and 10(2.7%) nonfatal stroke cases (Fig. 1). The patients were divided into four groups according to the quartiles of the GRACE risk score [Quartile 1 (≤ 122): n = 93; Quartile 2 (123–143): n = 92; Quartile 3 (144–165): n = 90; Quartile 4 (≥ 166): n = 90] and the baseline characteristics of the patients were analyzed (Table 1). Patients with a higher GRACE risk scores were more likely to be male, smoke cigarettes, drink alcohol and aging. The mean heart rate, mean blood pressure, the prevalence of MACE, Scr and BUN levels, and use of aspirin increased significantly with a higher GRACE risk score. The cardiac enzymes, total cholesterol and fasting glucose values, the prevalence of diabetes, hypertension, sepsis and chronic kidney disease, history of MI or PCI, use of antihypertensive drug and lipid-lowering drug, and other clinical examination reports did not differ among the GRACE risk score quartile groups.

**Table 1:** Characteristics of the NSTEMI Patients with MVD at Baseline

Characteristic	Quartile 1 (≤ 122)	Quartile 2 (123–143)	Quartile 3 (144–165)	Quartile 4 (≥ 166)	*P*-value
N	93	92	90	90	–
Men (%)	85(91.4%)	61(66.3%)	64(71.1%)	68(75.6%)	< 0.001
Age (y)	57(48–64)	65(57–72)	69(63–74)	74(71–79)	< 0.001
BMI (kg/m^2^)	24.8(22.9–27.7)	24.5(22.3–26.5)	24.0(21.3–26.4)	23.9(22.1–25.1)	0.060
Heart rate(bpm)	72(64–84)	73(66–81)	72(68–82)	78(70–92)	0.007
SBP (mmHg)	120(110–136)	129(115–144)	134(119–152)	134(124–148)	< 0.001
DBP (mmHg)	70(60–79)	72(65–80)	76(69–84)	80(70–92)	< 0.001
*Medical history*					
Cigarette use (%)	77(82.8%)	53(57.6%)	49(54.4%)	44(48.9%)	< 0.001
Uses alcohol (%)	36(38.7%)	24(26.1%)	18(20.0%)	16(17.8%)	0.005
Diabetes mellitus (%)	22(23.7%)	23(25.0%)	27(30.0%)	32(35.6%)	0.270
Hypertension (%)	46(49.5%)	58(63.0%)	48(53.3%)	53(58.9%)	0.258
Previous MI (%)	1(1.1%)	7(7.6%)	7(7.8%)	4(4.4%)	0.133
Previous PCI (%)	3(3.2%)	5(5.4%)	9(10.0%)	9(10.0%)	0.188
Stroke (%)	9(9.7%)	14(15.2%)	11(12.2%)	18(20.0%)	0.221
Chronic kidney disease (%)	1(1.1%)	3(3.3%)	3(3.3%)	2(2.2%)	0.731
Sepsis (%)	0	0	0	0	-
*Laboratory on admission*					
Troponin I	0.9(0.2–3.1)	1.0(0.4–3.7)	1.3(0.4–6.3)	1.2(0.3–4.0)	0.256
CK-MB (mmol/L)	10.4(3.1–51.8)	14.3(4.4–34.1)	18.4(5.5–43.5)	12.8(4.5–38.3)	0.503
WBC (× 10^9^/L)	8.5(6.9–10.7)	8.3(6.7–10.1)	8.2(6.6–10.1)	8.6(6.6–10.8)	0.877
C-reactive protein (mg/L)	3.4(0.9–8.3)	2.8(0.4–9.9)	2.0(0.4–15.0)	5.5(0.8–14.1)	0.236
AST (U/L)	45.0(25.2–76.6)	41.5(29.1–70.6)	46.1(25.6–82.7)	44.0(25.8–87.7)	0.951
ALT (U/L)	28.9(22.3–44.6)	27.2(19.9–39.9)	31.9(18.7–42.3)	25.0(18.1–43.9)	0.175
Scr (mmol/l)	70.3(62.0–85.7)	70.4(61.6–86.1)	76.3(65.8–88.9)	83.6(65.0–102.3)	0.006
BUN (mmol/l)	4.9(4.0–6.1)	5.3(4.2–6.8)	5.4(4.3–7.0)	6.5(4.9–8.0)	< 0.001
Fasting glucose (mmol/L)	5.9(5.1–7.2)	6.1(5.1–7.4)	6.1(5.2–8.5)	6.2(5.2–7.9)	0.265
Total cholesterol (mmol/L)	4.68(3.76–5.42)	4.56(3.96–5.39)	4.40(3.70–5.07)	4.37(3.60–4.88)	0.063
Triglycerides (mmol/L)	1.39(1.00–1.99)	1.43(1.16–2.19)	1.48(1.10–2.07)	1.62(1.20–2.78)	0.076
LDL cholesterol (mmol/L)	3.00(2.24–3.57)	2.88(2.26–3.72)	2.75(2.07–3.22)	2.83(2.17–3.14)	0.105
HDL cholesterol (mmol/L)	0.99(0.86–1.20)	1.03(0.92–1.20)	1.02(0.81–1.20)	1.00(0.90–1.23)	0.436
*Medication at follow up*					
Aspirin	93(100%)	92(100%)	86(95.6%)	90(100%)	0.006
Clopidogrel	29(31.2%)	25(27.2%)	24(26.7%)	35(38.9%)	0.257
Ticagrelor	64(68.8%)	66(71.7%)	64(71.1%)	56(62.2%)	0.497
Beta-blocker	52(55.9%)	56(60.9%)	58(64.4%)	44(48.9%)	0.170
ACE-inhibitor	38(40.9%)	36(39.6%)	31(34.4%)	35(38.9%)	0.827
Statins	92(98.9%)	91(98.9%)	90(100%)	89(98.9%)	0.804
*Coronary arteriography*					
Time of symptom onset to PCI(h)	19.0(4.1–54.2)	21.3(3.1–69.4)	23.8(2.7–75.7)	21.9(4.2–74.8)	0.822
SYNTAX score	18.0(13.5–25.0)	20.0(14.0–24.5)	21.5(13.8–28.0)	21.0(14.8–29.0)	0.080
TIMI flow grade before PCI					0.480
TIMI 0	16(17.2%)	26(28.3%)	27(30.0%)	23(25.6%)	
TIMI 1	10(10.8%)	5(5.4%)	3(3.3%)	6(6.7%)	
TIMI 2	8(8.6%)	12(13.0%)	6(6.7%)	5(5.6%)	
TIMI 3	59(63.4%)	49(53.3%)	54(60.0%)	56(62.2%)	
TIMI flow grade after PCI					0.797
TIMI 0	1(1.1%)	0	1(1.1%)	1(1.1%)	
TIMI 3	92(98.9%)	92(100%)	89(98.9%)	89(98.9%)	
Number of stents implanted	2(1–2)	2(1–2)	2(1–2)	2(1–2)	0.902
*Echocardiography*					
LVID (mm)	47(43–49)	45(42–50)	45(41–48)	49(43–53)	0.280
LVEF(%)	66(60–71)	65(59–69)	67(60–71)	63(57–68)	0.065
MACE	10(10.8%)	14(15.2%)	20(22.2%)	34(37.8%)	< 0.001

The data were presented as median and interquartile ranges or percentages, unless otherwise indicated. *ACE* angiotensin converting enzyme; *ALT* alanine aminotransferase; *AST* aspartate aminotransferase; *BMI* body mass index; *BUN* blood urea nitrogen; *CK* creatine kinase; *DBP* diastolic blood pressure; *GRACE* Global Registry of Acute Coronary events; *HDL* high-density lipoprotein; *LDL* low-density lipoprotein; *LVID* left ventricular internal diameter; *LVEF* left ventricular ejection fraction; *MACE* major adverse cardiovascular events; *PCI* percutaneous coronary intervention; *SBP* systolic blood pressure; *Scr* serum creatinine

### Association between GRACE risk score and MACE

**Fig. 2:**
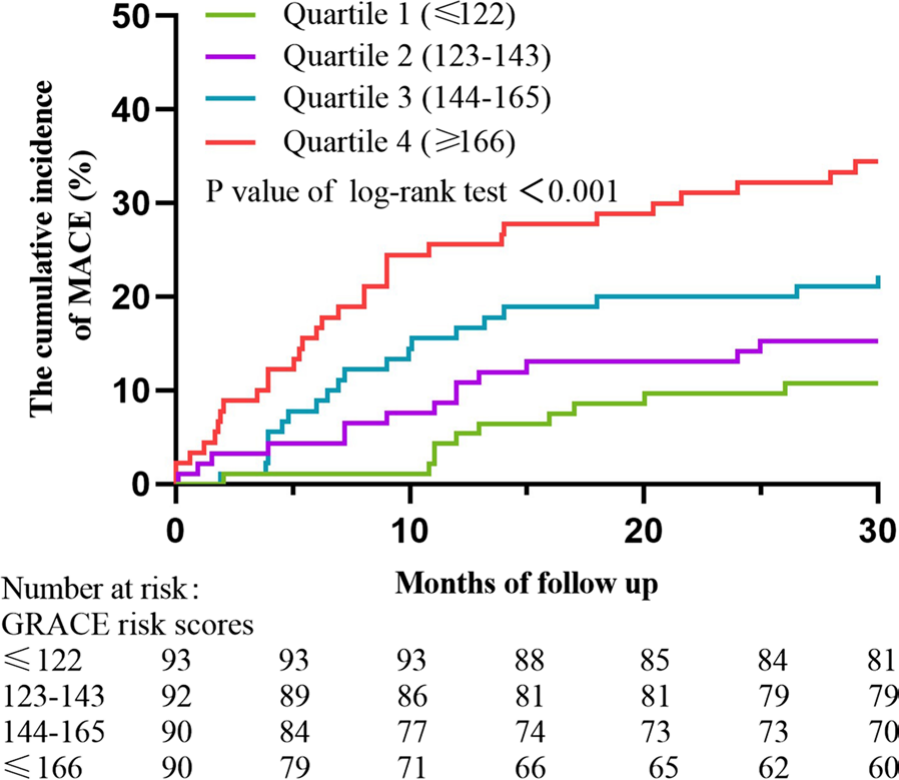
Kaplan-Meier plot of cumulative probability of cardiovascular events by quartiles of the GRACE risk scores. MACE, major adverse cardiovascular events

At 2.5 years, the cumulative incidence rates of the MACE were shown according to the GRACE risk score levels in Fig. 2, and the rates were significantly higher in the fourth quartile group than in the first quartile group (*P* < 0.001). To further explore the correlation between the GRACE score and the prevalence of MACE, Cox proportional hazards model analysis was performed. In univariable analyses, older age, higher heart rate on admission, higher BUN and Scr, higher GRACE risk score and SYNTAX score, history of diabetes and PCI, but not other medical history and other clinical examination reports, were associated with MACE (Additional file 1: Table S1). The age- and sex-adjusted HR increased linearly with elevating GRACE score levels, and this relationship remained significant after adjusting for age, sex, BMI, heart rate, systolic blood pressure, smoking status, alcohol use, diabetes, hypertension, chronic kidney disease, previous MI, previous PCI, BUN, Scr, and use of aspirin patients (Table 2). In the multivariable analyses model, the fourth quartile of GRACE score (≥ 166) was associated with increased risk of the MACE compared with the first quartile of GRACE score (≤ 122), after adjusting for the above confounding factors [HR 3.64, 95% confidence interval (CI) 1.32–10.01, *P* = 0.012]. Every 10-point increment in the GRACE score was similarly associated with an increased risk of the MACE, after adjusting for the confounding factors (HR 1.19, 95% CI 1.06–1.32, *P* = 0.002).

**Table 2:** Association between the GRACE risk scores and MACE

GRACE risk scores	Follow up period (months)	No. (%) of event	Age- and Sex-adjusted		Multivariable-adjusted*	
			HR (95% CI)	*P*-value	HR (95% CI)	*P*-value
Quartile 1 (≤ 122)	36(33–40)	10(10.8%)	1.00 (Reference)	-	1.00 (Reference)	-
Quartile 2 (123–143)	36(32–39)	14(15.2%)	1.30(0.55–3.06)	0.547	1.49(0.62–3.57)	0.374
Quartile 3 (144–165)	36(30–39)	20(22.2%)	1.87(0.80–4.36)	0.149	2.06(0.83–5.16)	0.121
Quartile 4 (≥ 166)	33(10–39)	34(37.8%)	3.27(1.37–7.84)	0.008	3.64(1.32–10.01)	0.012
Every 10-point increase in the GRACE risk scores	-	78(21.4%)	1.20(1.09–1.31)	< 0.001	1.19(1.06–1.32)	0.002

*Adjusted for age, sex, BMI, heart rate, systolic blood pressure, smoking, drinking, diabetes, hypertension, chronic kidney disease, previous MI, previous PCI, BUN, Scr, and use of aspirin patients. *BMI* body mass index; *BUN* Blood urea nitrogen; *CI* confidence interval; *GRACE* Global Registry of Acute Coronary events; *HR* hazard ratio; *MACE* major adverse cardiovascular events; *PCI* percutaneous coronary intervention; *Scr* serum creatinine

To avoid overfitting of the multivariable analyses model, the stepwise forward Cox regression model was performed to identify predictors based on the likelihood ratio test. The results showed that GRACE score (HR 1.014, 95% CI 1.006–1.021, *P* < 0.001), SYNTAX score (HR 1.101, 95%CI 1.070–1.133, *P* < 0.001), and BUN (HR 1.082, 95%CI 1.020–1.148, *P* = 0.009) were independently associated with MACE (Table 3).

**Table 3:** Forward stepwise Cox regression analysis for MACE in NSTEMI patients with MVD

Risk Factor	Hazard ratio	95% confidence interval	*P*-value
GRACE score	1.014	1.006–1.021	< 0.001
SYNTAX score	1.101	1.070–1.133	< 0.001
BUN	1.082	1.020–1.148	0.009

*BUN* Blood urea nitrogen; *MACE* major adverse cardiovascular events

### Estimation of the risk prediction ability for all-cause mortality and MACE

To investigate the predictive value of the GRACE score for all-cause mortality and MACE, receiver operating characteristic curve analysis were performed (Table 4). The results demonstrated that GRACE score (*c* = 0.72, 95% CI 0.59–0.85, *P* = 0.001), SYNTAX score (*c* = 0.75, 95% CI 0.66–0.84, *P* < 0.001), and BUN on admission (*c* = 0.78, 95% CI 0.66–0.89, *P* < 0.001) showed a good predictive value of all-cause mortality. For the prediction of comprehensive MACE, the discriminative value of GRACE score, and BUN decreased to varying degrees. However, the SYNTAX score maintained its predictive power in predicting MACE. Combining the three predictors significantly improved the performance of predicting cardiovascular events (all-cause mortality: *c* = 0.82, 95% CI 0.74–0.92, *P* < 0.001; MACE: *c* = 0.81, 95% CI 0.75–0.86, *P* < 0.001**)**.

**Table 4:** ROC curve analysis for the predictive value of models for all-cause mortality and MACE

Model	All-cause mortality		MACE	
	*c*-statistic (95% CI)	*P*-value	*c*-statistic (95% CI)	*P-*value
GRACE score	0.72(0.59–0.85)	0.001	0.69(0.62–0.75)	< 0.001
SYNTAX score	0.75(0.66–0.84)	< 0.001	0.75(0.70–0.81)	< 0.001
BUN	0.78(0.66–0.89)	< 0.001	0.69(0.62–0.76)	< 0.001
GRACE score, SYNTAX score, and BUN	0.82(0.74–0.92)	< 0.001	0.81(0.75–0.86)	< 0.001

*BUN* Blood urea nitrogen; *MACE* major adverse cardiovascular events

## Discussion

This study explored the relationship between the GRACE risk score and the occurrence of MACE in NSTEMI patients with MVD. In our study, we noticed a significant correlation between the occurrence of MACE and the GRACE risk score. Even after adjusting for confounders, the GRACE risk score was still independently associated with MACE. The GRACE risk score was previously reported for predicting the risk of all-cause mortality or (and) MI in patients with NSTEMI [6–9]. However, the value of the GRACE risk score for MACE in MVD remained undetermined. Our study found that the GRACE risk score performed well in predicting all-cause mortality in MVD, but was less powerful in predicting MACE. Furthermore, we also found that combining the GRACE risk score with the SYNTAX score, and BUN significantly improved the ability to predict MACE.

In our study, MVD accounted for 48% of NSTEMI patients. This finding was consistent with previous reports that a substantial proportion (up to 50%) of patients with NSTEMI undergoing invasive management had MVD on the angiography [1–3, 13]. Compared with single-vessel disease, MVD had a worse quality of life and higher cardiovascular risk [4, 14]. Patients with MVD were usually accompanied with comorbid risk factors such as elderly age, hypertension, diabetes impaired renal function, as well as lung disease, a higher likelihood of a previous MI and dyslipidemia [4, 15]. Therefore, it was very important to provide long-term MACE risk assessment in NSTEMI patients with MVD for secondary prevention.

The multiple risk factors make it challenging to evaluate the long-term prognosis and risk stratification of MVD to allow early intervention. Among many prognostic models aimed to estimate the risk of all-cause mortality, the GRACE risk score provided the best discriminative power. [7, 8] Although many validation studies confirmed the validity of GRACE in multiple clinical settings, to our knowledge, we were the first to evaluate its performance in predicting long-term outcomes in patients with MVD. The GRACE score, which was recommended for predicting all-cause mortality in NSTEMI patients, did not show a particular advantage in predicting MACE performance compared with other risk factors in our study. This result may be due to differences in the predictive value of the GRACE score between different clinical endpoints [7, 16, 17]. The GRACE score prospectively verified that the *c*-statistic value of ACS patients predicting death 6 months after admission was 0.81, while the *c*-statistic value predicting death or MI was 0.73 [18]. This may be an important reason why the difference in the GRACE score predicts all-cause mortality (*c* = 0.72) and MACE (*c* = 0.69) of NSTEMI patients with MVD.

All versions of the GRACE risk score model used the same eight variables (age, systolic blood pressure, heart rate, Scr, cardiac arrest at admission, elevated cardiac biomarkers, ST-segment deviation, and Killip class) for risk prediction [9]. The eight variables were transient indicators at admission, without considering the patient’s comorbidity or long-term indicators (such as the degree of coronary atherosclerosis) [7]. The early death of NSTEMI patients was more attributable to ischemia/thrombosis-related events, and the later death was more related to the progression of atherosclerosis and non-cardiovascular causes such as diabetes [4, 9]. This also explained the good performance of diabetes and SYNTAX score in predicting all-cause mortality in patients with MVD. Similarly, the inadequate predicting value of the GRACE score for long-term prognosis of patients with MVD may be due to comorbidity and coronary atherosclerosis which are not taken into consideration. Recently, a study found that in predicting the severity and extent of coronary artery stenosis, GRACE score can detect normal coronary individuals or mild CAD patients very well. But in high-risk patients it had a high negative predictive value [19]. In patients with MVD, the severity and extent of coronary artery stenosis is an important factor to the MACE. Thus, this may also a reason for the inadequate predicting value of the GRACE score for long-term prognosis of patients with MVD.

SYNTAX score and BUN were also closely associated with the occurrence of MACE in our forward stepwise Cox regression analysis. SYNTAX score was a risk scoring method for quantitatively evaluating the complexity of lesions based on 11 anatomical features of coronary angiographic lesions [12, 20]. Compared with GRACE scores, SYNTAX score provided an effective, objective, evidence-based tool to evaluate the severity and extent of coronary artery stenosis [21]. For patients with MVD who were candidates for both PCI and coronary artery bypass grafting (CABG), the SYNTAX score can be used to help make treatment decisions [12]. In patients with three-vessel disease undergoing PCI and stenting, a higher SYNTAX scores significantly predicted a higher risk of MACE [22]. Consistent with previous studies [12, 21, 23], we also found that the SYNTAX score had good performance in predicting all-cause mortality and MACE in our study. Besides, the BUN levels were strongly associated with cardiovascular events in patients with MVD in our study. This finding was consisted with the previous study, in patients with PCI when GFR < 30 mL/min people were more susceptible to occurrence of MACE [24]. BUN is a predictor of the prognosis of patients with heart failure, and the correlated better with mortality than did Scr and glomerular filtration rate [25–27]. This may explain why BUN was related to MACE in the Cox analysis, but Scr showed a non-significant P-value in the multivariate analysis. Finally, we found that the GRACE risk score combined with the SYNTAX score, and BUN improved the ability to predict all-cause mortality (*c* = 0.82) and MACE (*c* = 0.81) to a satisfactory level. This suggested that BUN was an important predictor of long-term prognosis in patients with MVD, which may provide an effective tool for establishing an ideal model for predicting cardiovascular events.

This study had several limitations. Firstly, it was a single center study with relatively small sample size. Secondly, we were unable to assess differences in GRACE scores due to improved medical conditions. Finally, we were not able to estimate thresholds for each event because of the small number of secondary outcome events. Therefore, further studies with multicenter and larger samples are necessary.

Despite these limitations, the conclusions of our study are still reliable. Based on the assumptions of a two-sided alpha of 5%, 80% power, and a 20% dropout rate, the estimated sample size according to the Freedman’s method was 329, while the sample size for this study was 365 [28]. In addition, we adjusted for a variety of clinical risk factors and potential confounders that could confound interpretation of the data.

In conclusion, the GRACE score showed an acceptable predictive value for all-cause mortality in patients with MVD, whereas it was insufficiently effective in predicting MACE. BUN on admission was independent predictor of the MACE in patients with MVD. Our finding supported that BUN may be potentially useful in predicting the long-term prognosis of NSTEMI patients with MVD.

## Supplementary Information

**Additional file 1**. Supplemental information.

## Data Availability

The datasets used and/or analyzed during the current study available from the corresponding author on reasonable request.
