# Supplementary material for: The prognostic utility of GRACE risk score in predictive adverse cardiovascular outcomes in patients with NSTEMI and multivessel disease
Source: BMC Cardiovasc Disord. 2022 Dec 26;22:568. doi: 10.1186/s12872-022-03025-6 (PMC9791745; doi:10.1186/s12872-022-03025-6)
Supplement: Supplementary file 1 — Additional file 1. Supplemental information. [file 12872_2022_3025_MOESM1_ESM.docx]

**Table S1**. Univariate cox regression analysis for the MACE.

| **Characteristic** | **Hazard ratio** | **95% confidence interval** | **P- value** |
| --- | --- | --- | --- |
| Sex(men) | 0.866 | 0.521-1.440 | 0.580 |
| Age (y) | 1.041 | 1.018-1.064 | 0.001 |
| BMI (kg/m^2^) | 0.944 | 0.882-1.011 | 0.100 |
| Heart rate(bpm) | 1.003 | 1.000-1.006 | 0.032 |
| SBP (mmHg) | 0.996 | 0.986-1.007 | 0.498 |
| DBP (mmHg) | 0.987 | 0.969-1.006 | 0.174 |
| GRACE score | 1.019 | 1.012-1.026 | ＜0.001 |
| **Medical history** |  |  |  |
| Smoking | 1.069 | 0.675-1.692 | 0.776 |
| Drinking | 0.703 | 0.406-1.218 | 0.209 |
| Diabetes mellitus | 2.851 | 1.828-4.446 | ＜0.001 |
| Hypertension | 1.425 | 0.897-2.263 | 0.134 |
| Previous MI | 1.207 | 0.488-2.988 | 0.684 |
| Previous PCI | 2.332 | 1.232-4.413 | 0.009 |
| Stroke | 1.157 | 0.625-2.140 | 0.642 |
| Chronic kidney disease | 1.169 | 0.287-4.762 | 0.827 |
| **Laboratory on admission** |  |  |  |
| Troponin I | 0.999 | 0.985-1.013 | 0.880 |
| CK-MB (mmol/L) | 0.999 | 0.995-1.003 | 0.570 |
| WBC (×10^9^/L) | 1.056 | 0.989-1.128 | 0.104 |
| C-reactive protein (mg/L) | 1.009 | 1.002-1.017 | 0. 060 |
| AST (U/L) | 1.000 | 0.999-1.002 | 0.581 |
| ALT (U/L) | 0.995 | 0.985-1.004 | 0.278 |
| Scr (mmol/l) | 1.007 | 1.004-1.010 | ＜0.001 |
| BUN (mmol/l) | 1.132 | 1.082-1.185 | ＜0.001 |
| Fasting glucose (mmol/L) | 1.085 | 1.015-1.159 | 0.061 |
| Total cholesterol (mmol/L) | 0.963 | 0.791-1.172 | 0.704 |
| Triglycerides (mmol/L) | 0.963 | 0.829-1.120 | 0.625 |
| LDL cholesterol (mmol/L) | 0.869 | 0.676-1.117 | 0.274 |
| HDL cholesterol (mmol/L) | 1.445 | 0.765-2.729 | 0.256 |
| **Medication at follow up** |  |  |  |
| Aspirin | 0.893 | 0.537-1.485 | 0.663 |
| Clopidogrel | 1.092 | 0.682-1.748 | 0.715 |
| Ticagrelor | 1.003 | 0.623-1.614 | 0.991 |
| Beta-blocker | 1.011 | 0.645-1.585 | 0.960 |
| ACE-inhibitor | 0.848 | 0.532-1.352 | 0.488 |
| Statins | 0.642 | 0.089-4.619 | 0.660 |
| **Coronary arteriography** |  |  |  |
| Onset to primary PCI(h) | 1.000 | 0.996-1.004 | 0.895 |
| SYNTAX score | 1.115 | 1.084-1.147 | ＜0.001 |
| Stent number implanted | 1.026 | 0.807-1.305 | 0.831 |
| **Echocardiography** |  |  |  |
| LVID (mm) | 0.981 | 0.935-1.029 | 0.435 |
| LVEF | 0.994 | 0.969-1.019 | 0.643 |

The data were presented as median and interquartile ranges or percentages, unless otherwise indicated. ACE, angiotensin converting enzyme; ALT, alanine aminotransferase; AST, aspartate aminotransferase; BMI, body mass index; BUN, blood urea nitrogen; CK, creatine kinase; DBP, diastolic blood pressure; GRACE, Global Registry of Acute Coronary events; HDL, high-density lipoprotein; LDL, low-density lipoprotein; LVID, left ventricular internal diameter; LVEF, left ventricular ejection fraction; MACE, major adverse cardiovascular events; PCI, percutaneous coronary intervention; SBP, systolic blood pressure; Scr, serum creatinine.
